# An endpoint multiplex PCR/RT-PCR for detection of viral agents of bovine respiratory disease

**DOI:** 10.1007/s42770-026-02097-2

**Published:** 2026-11-02

**Authors:** Natália Hettwer Pedroso Alessio, José Valter Joaquim Silva Júnior, Iasmim Flôres Dionisio, Vinicius Soares dos Santos, Viviani Gomes, Rudi Weiblen, Eduardo Furtado Flores

**Affiliations:** 1 Setor de Virologia, Departamento de Medicina Veterinária Preventiva, Universidade Federal de Santa Maria, Av. Roraima, 1000, Prédio 63 A, Camobi, Santa Maria, Rio Grande do Sul 97105-900 Brazil; 2 Programa de Pós-graduação em Medicina Veterinária, Universidade Federal de Santa Maria, Santa Maria, Rio Grande do Sul Brazil; 3 Departamento de Microbiologia e Parasitologia, Universidade Federal de Santa Maria, Santa Maria, Rio Grande do Sul Brazil; 4 Laboratório NB3 de Neuroimunologia, Universidade Federal de Santa Maria, Santa Maria, Rio Grande do Sul Brazil; 5 Programa de Pós-graduação em Farmacologia, Universidade Federal de Santa Maria, Santa Maria, Rio Grande do Sul Brazil; 6 Setor de Virologia, Instituto Keizo Asami, Universidade Federal de Pernambuco, Pernambuco, Brazil; 7 Departamento de Clínica Médica, Faculdade de Medicina Veterinária e Zootecnia, Universidade de São Paulo, São Paulo, Brasil

**Keywords:** Multiplex polymerase chain reaction, Clinical laboratory techniques, Bovine viral diagnosis, Bovine diarrhea virus 1, Bovine diarrhea virus 2, Bovine herpesvirus 1, Bovine parainfluenza 3, Bovine coronavirus

## Abstract

Bovine respiratory disease (BRD) is a clinicopathological syndrome caused by complex interactions among host and environmental factors, viruses and bacteria. The multi-agent BRD etiology makes laboratory diagnosis challenging, as various pathogens should be investigated for an accurate results. Herein, we describe an endpoint multiplex PCR/RT-PCR for detection of major BRD-associated viruses: bovine herpesvirus 1 (BoAHV-1), bovine pestiviruses (BVDV-1, BVDV-2, HoBiPeV), bovine parainfluenza virus 3 (BPIV-3), bovine respiratory syncytial virus (BRSV) and bovine coronavirus (BCoV). We designed high-coverage and target-specific primers to be properly combined in the multiplex assay. Following standardization of individual reactions, the multiplex PCR/RT-PCR was optimized for reaction conditions and mix composition. The amplicons of the five targets were easily differentiated in agarose gel: 121 bp (BPIV-3), 157 bp (BoAHV-1), 204 bp (pestiviruses), 282 bp (BRSV) and 323 bp (BCoV). The limits of detection (LoDs) of our multiplex were 38 TCID_50_ (BoAHV-1 and BPIV-3), 16 TCID_50_ (pestiviruses), 13 TCID_50_ (BRSV) and a 10^− 2^ dilution of a pool of five BCoV positive samples. No amplification of the main bacteria (*Pasteurella multocida*; *Mannheimia haemolytica* and *Histophilus somni*) potentially involved in BRD was observed. Finally, 289 respiratory specimens from animals with respiratory/nasal signs were evaluated by individual and multiplex PCR/RT-PCR tests to assess the diagnostic performance of the assay. Both tests resulted in 14.8% positive samples (43/289) being 19 for BPIV-3 (19/43), ten for BCoV (10/43), seven for BRSV (7/43), six for BoAHV-1 (6/43) and one for pestiviruses (1/43). Overall, our multiplex PCR/RT-PCR assay represents an alternative for an easier, faster and more practical laboratory diagnosis of viruses associated with BRD.

**Supplementary Information:**

The online version contains supplementary material available at https://doi.org/10.1007/s42770-026-02097-2.

## Introduction

Bovine respiratory disease (BRD) is a complex, multifactorial and multiagent disease affecting primarily the respiratory tract of young cattle in dairy and beef feedlot systems worldwide [1, 2]. The economic impacts of BRD are mainly associated with decreased productivity, mortality, treatment, vaccination and labor costs [3, 4].

The etiology of BRD involves interactions among host, environmental and pathogen factors, notably associated with viral and bacterial infections. Briefly, the main bacterial agents involved in BRD etiology include *Pasteurella multocida*,* Histophilus somni*, *Mannheimia haemolytica* and *Mycoplasma bovis.* The major viral agents associated with BRD are bovine alphaherpesvirus 1 (BoAHV-1), bovine viral diarrhea virus (BVDV), bovine parainfluenza virus 3 (BPIV-3), bovine respiratory syncytial virus (BRSV) and bovine coronavirus (BCoV) [5–8]. BoAHV-1 is an enveloped virus with a double-stranded DNA (dsDNA) genome of the family *Orthoherpesviridae*, genus *Varicellovirus* and species *Varicellovirus bovinealpha1* [9]. Bovine viral diarrhea viruses (BVDV) are enveloped viruses with a highly variable single-stranded RNA genome (ssRNA), belonging to the family *Pestiviridae*, genus *Orthopestivirus*, and encompasses two viral species, *Orthopestivirus bovis* (BVDV-1) and *Orthopestivirus tauri* (BVDV-2). A third bovine pestivirus, *Orthopestivirus brazilense* (HoBiPeV), has also been associated with respiratory disease in cattle [9–11]. BPIV-3, an enveloped virus with ssRNA genome, belonging to the family *Paramyxoviridae*, genus *Respirovirus* and species *Respirovirus bovis* [9], is a viral agent extensively associated with BRD [9, 12]. BRSV is an enveloped virus with a ssRNA genome belonging to the family *Pneumoviridae*, genus *Orthopneumovirus* and species *Orthopneumovirus bovis* [9]. BCoV (ssRNA genome), family *Coronaviridae*, genus *Betacoronavirus* and species *Betacoronavirus gravedinis* [9], has been also detected in cases of respiratory or enteric disease in cattle [6, 12–14].

The field diagnosis of BRD is usually based on the clinical presentation [15], an approach with limitations, considering subclinical or atypical cases and those with nonspecific clinical signs. Thus, laboratory diagnosis is necessary to provide the etiological cause(s) of the disease [16]. The complex nature of BRD, in turn, impacts laboratory diagnosis in several ways [1]. For example, the etiological diagnosis may be underestimated, costly and time-consuming when tests are performed for individual agents, such as viral isolation, PCR, RT-PCR, serology and microbiological culture [17, 18]. In contrast, multiplex molecular assays are capable of detecting different combinations of pathogens simultaneously, providing a comprehensive and more efficient laboratory diagnosis. However, most assays available for BRD diagnosis are based on real-time technology (qPCR), which requires specialized and expensive equipment and probes, increasing the overall cost of testing and potentially limiting their implementation in resource-limited settings [5, 8, 19–22].

Herein, we describe a multiplex endpoint PCR/RT-PCR assay for detecting the main viral agents associated with BRD: BoAHV-1, bovine pestiviruses (BVDV-1, BVDV-2 and HoBiPeV), BRSV, BPIV-3 and BCoV. Overall, our assay showed adequate sensitivity and specificity, representing a useful and accessible tool for simultaneous and differential diagnosis of the main viral agents of BRD. Its major advantage is the combination of a cost-effective endpoint multiplex PCR/RT-PCR format with high-coverage primers, providing an affordable alternative for routine veterinary diagnostic laboratories, particularly those without access to real-time PCR platforms.

## Materials and methods

### Study design

Initially, primer sets with high coverage and specificity were selected from the literature or designed for the main viral agents of BRD. Next, individual PCR/RT-PCR were standardized for each target. Subsequently, the multiplex assay was standardized by optimizing reaction and mix conditions. Then, the analytical sensitivity (limit of detection, LoD) and specificity of the multiplex PCR/RT-PCR were determined. In addition, the LoDs were also determined in bovine samples spiked with positive controls to investigate a potential influence of the biological matrix on the assay performance. Finally, nasal swabs and lung tissue from animals with BRD were tested by the multiplex and the results compared with those of individual PCR/RT-PCR.

### Primer design

The following criteria were used for primer selection: high coverage (based on *in silico* analysis of genomic regions or complete/near-complete genomes deposited in GenBank), dimer formation (https://idtdna.com/pages/tools/oligoanalyzer), melting temperature, specificity [Basic Local Alignment Search Tool (BLAST), https://blast.ncbi.nlm.nih.gov/Blast.cgi] and amplicon size for correct target differentiation. For this, a database was created from GenBank for each viral target with a total of 58 (BoAHV-1), 69 (BRSV) and 28 (BPIV-3) sequences of complete/near-complete genomes. For BVDV-1 (159), BVDV-2 (296) and HoBiPeV (134) sequences of the 5′-untranslated region (5′-UTR) were used. In this study, primer sets that annealed to all sequence in the database, allowing up to two nucleotide mismatches (2 nt) per primer, were selected. In addition, non-specific annealing with other BRD-associated targets was evaluated (BLAST).

The selected primers and the respective amplicons used in the multiplex assay are presented in Table 1. Primers were selected to maximize inclusivity across the genetic diversity currently available in GenBank, based on all publicly available sequences without restrictions regarding year of collection, lineage, or geographic origin, while maintaining target specificity and providing broad expected coverage of circulating strains/genotypes of each viral target.

**Table 1:** Primer sets and limits of detection (LoD) for each target in the PCR/RT-PCR multiplex

Primers	Sequence (5’−3’)	Melting temperature (°C)^a^	Agent (target)	Amplicon (bp)	Reference	LoD
PIV3-BN CE strain-2819 F	GAGGACACAGAAGAGAGCACTC	52.1	BPIV-3 (P)^b^	121	This study	38 TCID_50_
PIV3-BN CE strain-2939R	CCACACAYACAACTCTCTTGTCTTG	55.1				
BoAHV 1/5_UL15_F	CCTTGAGGTTGTAGAGGAAGC	55.7	BoAHV-1/BoAHV-5 (UL15)^c^	157	This study	38 TCID_50_
BoAHV- 1/5_UL15_R	GACTTCAACCTCCTCTTCGT	54.7				
^*^BVDV_Uni_5UTR_F	GGDAGTCGTCARTGGTTCGAC	56.2	BVDV-1 BVDV-2 HoBiPeV (5’UTR)^d^	204	This study	16 TCID_50_
^*^BVDV_Uni_5UTR_R	TCCATGTGCCATGTACAGCAG	58.3				
BRSV_N_F2	TGCTATGTGTTGCTGCTTTGGTTATAAC	57.2	BRSV (N)^e^	282	This study	13 TCID_50_
BRSV_N_R	CTGCTCCATATGCATTCATGAATAAC	50.5				
BECoV_1a_F3	TTGTGGTGCTTAATGCCCAC	55.7	BCoV (ORF1a)^f^	323	Pedroso et al., 2023	10^− 2^ pool of samples
BECoV_1a_R3	CCATCAGCAGAAAGTGCATGTG	52.5				

^a^ Calculated at https://www.idtdna.com/calc/analyzer (for 50 mM monovalent salt, Na^+^)

^b^ Phosphoprotein gene

^c^ Unique Long 15

^d^ Untranslated Region

^e^ Nucleoprotein gene

^f^ Open Reading Frame

*Primers designed using as reference the primers described by Monteiro et al. [23], with additional nucleotides to increase the melting temperature and advantage multiplex performance

### Positive controls

Strains of BoAHV-1 (Cooper), bovine pestiviruses (BVDV-1, Singer strain; BVDV-2, VS-253 and HoBiPeV, SV757/15) (all from the Virology Section, SV-UFSM), BPIV-3 (provided by Biovet/Vaxxinova), BRSV (Biovet/Vaxxinova) and BCoV (provided by Dr. Paulo Brandão, Universidade de São Paulo, USP) were used to standardize the individual PCR/RT-PCR reactions and the multiplex assay. Nucleic acid (RNA and DNA) extraction from cell culture supernatants inoculated with the respective viruses was performed simultaneously using a TRIzol™ (Invitrogen™)-based protocol, previously described by Pedroso et al. [24].

### Sequencing

After PCR amplification, the amplicons were purified with PureLink PCR Purification (Invitrogen™), according to the manufacturer’s instructions and subjected to nucleotide sequencing by the Sanger method, using the BigDye™ Terminator v3.1 Cycle Sequencing Kit (ThermoFischer Scientific™).

### PCR/RT-PCR panel

A panel of five individual reactions (for each viral target) was used as a reference assay to define true positive and true negative samples during the development of the assay. The reactions were standardized using the Taq DNA Polymerase, Recombinant kit (Thermo Fisher Scientific) for DNA targets and the RT-PCR was optimized using the two-step methodology, using random primers for the synthesis of complementary DNA (cDNA) (GoScript™ Reverse Transcription Mix, Random Primers, Promega) for RNA targets. In both cases, the PCR reaction conditions were optimized for primer annealing temperature and MgCl_2_ concentration.

###  Multiplex PCR/RT-PCR assay

The multiplex PCR/RT-PCR assay was developed to amplify BoAHV-1, bovine pestiviruses (BVDV-1, BVDV-2 and HoBiPeV), BRSV, BPIV-3 and BCoV sequences. Reactions were performed with the AgPath-ID™ One-Step RT-PCR kit (Applied Biosystems™). Mix conditions were optimized regarding primer concentration (0.1 µM, 0.2 µM, or 0.4 µM) and enzyme (0.6 µL, 1 µL or 1.2 µL). Reaction conditions were optimized regarding denaturation (95 °C–94 °C), annealing and extension temperatures (52–62 °C at 2 °C intervals) and times (15, 30, and 45 s) to favor amplification of all targets. Additionally, amplification protocols using 35 and 40 cycles were evaluated.

###  Analytical sensitivity and specificity of the multiplex PCR/RT-PCR

The LoD for each multiplex PCR/RT-PCR target was defined as the minimum TCID_50_ [tissue culture infection dose, Reed and Muench [25]] detected by the assay. Briefly, nucleic acids (DNA/RNA) extracted from culture supernatants with a known TCID_50_/mL were diluted ten-fold and submitted to PCR/RT-PCR multiplex. For BCoV, the LoD was assessed by diluting a pool of positive samples, as a viral isolate was unavailable. To assess the specificity of the multiplex assay, nucleic acids of other potential BRD targets were applied to the multiplex. All targets were initially tested by individual PCR assays using reactions standardized previously by our group (Pedroso, NH, unpublished), including *Pasteurella multocida* (provided by Dr. Juliana Felipetto Cargnelutti, UFSM), *Histophilus somni* (provided by Dr. Andrea Moreno, USP) and *Mannheimia haemolytica* (BOVILIS^®^ VISTA^®^ ONCE SQ - MSD Animal Health).

###  Analytical sensitivity of multiplex assay in spiked biological samples

Swab samples negative for the viral targets received at the diagnostic service of the SV-UFSM were spiked with previously quantified positive controls. Briefly, samples were centrifuged (1,190 x *g* for 10 min) and the supernatant was collected (100 µL). Subsequently, the supernatant was mixed with previously quantified viral suspensions (100 µL) and submitted to RNA/DNA extractions as described above. The LoD of detection in spiked samples for each target was calculated in the PCR/RT-PCR multiplex assay.

### Multiplex PCR/RT-PCR diagnostic performance

Respiratory tract samples (*n* = 289) were processed to assess the diagnostic performance of the multiplex assay. The sample set comprised 277 nasal swab samples originating from Holstein dairy calves on 16 farms at São Paulo state, Brazil, where cattle with clinical signs compatible with BRD were present (provided by Viviani Gomes, USP) and 12 samples received at SV-UFSM (10 lung tissues and 2 nasal swabs). All samples were stored at −80 °C until processing. Sample screening was performed with DNA and RNA extractions in pools of five samples, using the PureLink™ Viral RNA/DNA Mini Kit and application on the individual virological panel.

Next, samples from the positive pools were processed individually and subjected to RNA/DNA extraction using an adapted protocol based on TRIzol™ (Invitrogen™), previously described by Pedroso et al. [24]. Briefly, after the chloroform treatment step, the DNA phase was collected, washed twice with chloroform: isoamyl alcohol (24:1, v/v) and then added to the RNA phase. The following steps were performed according to the manufacturer’s protocol. DNA/RNA was eluted in 40 µL of Tris-EDTA (10 mM Tris-HCl, 1 mM EDTA, pH 8.0). The RNA/DNA concentration and quality were evaluated in a NanoDrop Lite Spectrophotometer (Thermo Scientific). The nucleic acid from individual samples was applied to both the virological panel and multiplex assay simultaneously and positive/negative results of each assay were recorded.

## Results

### Optimization PCR/RT-PCR panel

The standardization of the PCR/RT-PCR panel (individual reactions) was performed using Taq DNA Polymerase Recombinant (Thermo Fisher Scientific) with a final volume of 10 µL: 1 µL of cDNA/DNA, 0.4 µL of each primer (both at 10 µM), 0.5 µL of MgCl_2_ (50 mM), 0.2 µL of dNTPs (10 mM), 1 µL of buffer (10X), 0.1 µL of Taq DNA Polymerase (5U/µL) and 6.4 µL of nuclease-free water. The PCR reaction conditions followed the manufacturer’s recommendations for all targets and the annealing/extension temperature was standardized as 52 °C/45 s for amplification of BPIV-3, 54 °C/45 s for BoAHV-1, 58 °C/45 s for BRSV and BCoV, 62 °C/45 s for pestiviruses.

### Optimization of the multiplex PCR/RT-PCR assay

**Fig. 1:**
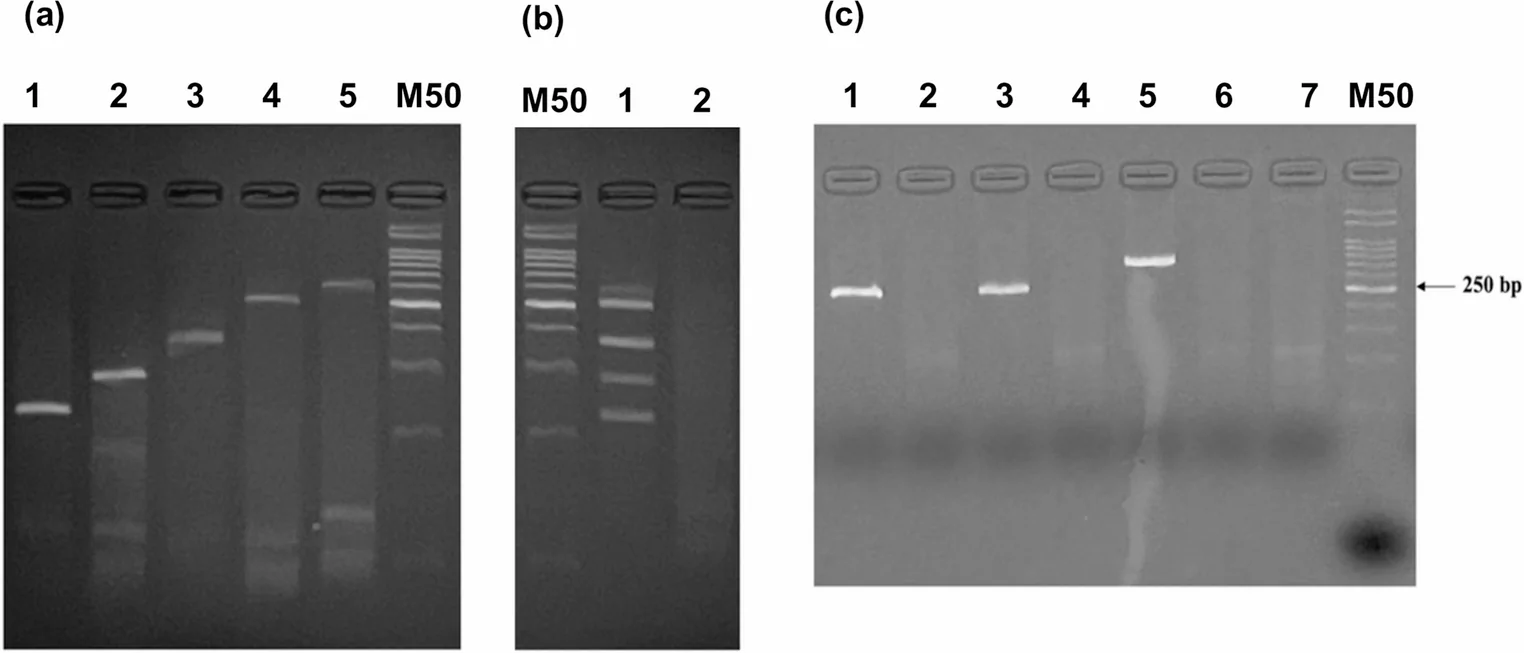
Endpoint multiplex PCR/RT-PCR. (**a**) Products of the viral multiplex PCR/RT-PCR assay with individual targets resolved on agarose gel (5%) stained with GelRed. 1: Positive control for BPIV-3 (121 bp); 2: BoAHV-1 (157 bp); 3: BVDV-1 (204 bp); 4: BRSV (282 bp); 5: BCoV (323 bp); M50: 50 bp DNA Ladder (marker) (Ludwig, RS, Brazil). (**b**) Products of the viral multiplex PCR/RT-PCR assay with simultaneous amplification of all targets resolved on agarose gel (5%) stained with GelRed. 1: Positive control for the five targets; 2: Negative control (nuclease-free water); M50: 50 bp DNA Ladder (marker). (**c**) Multiplex analytical specificity test for *H. somni*, *M. haemolytica* and *P. multocida.* 1, 3, 5: individual reaction to *H. somni* (219 bp), *M. haemolytica* (266 bp) and *P. multocida* (388 bp), respectively; 2, 4 and 6: multiplex reactions with *H. somni*, *M. haemolytica* and *P. multocida*, respectively; 7: negative control (nuclease-free water); M50: 50 bp DNA Ladder (marker). In (**a**), (**b**) and (**c**), amplicon migrations were slightly influenced by the GelRed stain (Biotium)

A multiplex PCR/RT-PCR assay for amplification of BoAHV-1, bovine pestiviruses (BVDV-1, BVDV-2 and HoBiPeV), BRSV, BPIV-3 and BCoV was standardized. The reaction was optimized to a final volume of 15 µL: 7.5 µL of 2X RT-PCR buffer, 1 µL of Taq Enzyme Mix, 0.12 µL of forward primers (50 µM) and 0.12 µL of reverse primers (50 µM), 0.3 µL of nuclease-free water and 5.0 µL of DNA and/or RNA. The reaction conditions were as follows: 45 °C for 10 min (reverse transcription), 95 °C for 10 min (initial denaturation and reverse transcriptase inactivation), followed by 35 cycles of denaturation at 95° C for 15 s, annealing/extension at 62 °C for 45 s. The PCR products were best resolved on 5% agarose gels. Figure 1 illustrates the products of the multiplex PCR/RT-PCR assay and the individual reaction of each target resolved on a 5% agarose gel and stained with GelRed. The identity of the agents was confirmed by nucleotide sequencing of the amplicons (data not shown).

### Sensitivity and specificity of multiplex PCR/RT-PCR assay

The LoDs for the targets of the PCR/RT-PCR multiplex assay were: 38 TCID_50_ for BoAHV-1 and BPIV-3, 16 for pestiviruses (BVDV-1, BVDV-2 and HoBiPeV), 13 for BRSV and a 10^− 2^ dilution pool of five positive samples for BCoV (Table 1). *H. somni*,* M. haemolytica* and *P. multocida* were not amplified in the viral multiplex assay and no nonspecific amplifications were observed during assay standardization (Fig. 1c). Additionally, non-template controls were included after the reverse transcription step and in the RT-PCR amplification reactions, and no amplification was observed in either control.

###  Sensitivity of the multiplex assay on spiked samples

The LoDs for bovine nasal swab samples spiked with viral targets were the same as those obtained for viral suspensions, *i*.*e*., 38 TCID_50_ for BoAHV-1 and BPIV-3, 16 for BVDV-1, BVDV-2 and HoBiPeV and 13 for BRSV. The LoD for BCoV on spiked samples was not performed because the analytical sensitivity of the assay had already been calculated on a pool of clinical samples.

### Diagnostic performance of the multiplex PCR/RT-PCR

We analyzed 289 clinical samples of BRD, including nasal swabs (*n* = 279) and lung tissue (*n* = 10). The multiplex assay was able to reproduce all the results of the individual viral panel and non-specific amplifications were not observed. Both trials resulted in 14.8% positive samples (43/289). Both assays confirmed 19 BPIV-3 positive samples (19/43), ten for BCoV (10/43), seven for BRSV (7/43), six for BoAHV-1 (6/43) and one positive for pestiviruses (1/43). All targets were detected in single infections and no mixed infections were detected.

## Discussion

The diagnosis of diseases potentially associated with multiple infectious agents, such as BRD, represents a challenge and is frequently frustrating. The use of individual tests for investigating each potential pathogen is time-consuming and costly, potentially hindering the identification of the full spectrum of agents involved.

Therefore, the use of tests encompassing more agents, as long as they are adequately sensitive and specific, is highly desirable for achieving reliable results [15]. In this context, multiplex molecular assays are timely alternatives since they allow a comprehensive diagnosis of several potential targets involved. Considering these issues, we developed an endpoint multiplex PCR/RT-PCR assay for simultaneous and differential detection of the main viruses associated with BRD: BoAHV-1, bovine pestiviruses (BVDV-1, BVDV-2 and HoBiPeV), BRSV, BPIV-3 and BCoV. Although other viruses may be occasionally involved in BRD, these targets are the most frequently detected [12].

Several experimental or commercial multiplex assays have been described for BRD diagnosis [20, 26]. For example, a qPCR/RT-qPCR assay was developed for detection of five viral BRD pathogens (BVDV, BoAHV-1, BRSV, BPIV3 and influenza D virus) [22]. However, the cost of qPCR assays is high due to the need for specific probes for each target, which can make them inaccessible to some laboratories. In contrast, conventional PCR assays are more cost-effective; however, they provide only qualitative detection, are generally less sensitive than qPCR assays, and relatively few assays have been described. For instance, a multiplex endpoint RT-PCR/PCR for detection of BoAHV-1, BVDV, *Chlamydia* spp. and *Mycoplasma* spp. was previously described [27].

Herein, we present an accessible, specific and sensitive endpoint PCR/RT-PCR assay targeting the five major BRD pathogens. Notably, the developed assay employs primers designed from representative and up-to-date datasets, ensuring high coverage, which would contribute to the performance of the multiplex. The combined DNA/RNA extraction previously described in our laboratory facilitates the extraction process while preserving the quality of the material [24]. The use of conventional PCR, in spite of some restrictions (*e*.*g*., time and labor), makes the protocol more affordable for many laboratories while keeping adequate sensitivity. Finally, the inclusion of the most frequent viruses associated with BRD worldwide ensures a broad coverage.

The analytical sensitivity of the multiplex was determined using positive controls for all individual agents previously quantified, demonstrating satisfactory amplification of all targets analyzed. Regarding BCoV, we did not have access to quantified viral isolates/strains. Nonetheless, our multiplex was able to detect the viral genome target up to a 10^− 2^ dilution from a pool of five positive samples, which we believe is a satisfactory result for an endpoint molecular multiplex. Furthermore, our assay also showed adequate specificity for use in laboratory diagnostics, not amplifying bacterial agents potentially related to BRD (*H. somni*,* M. haemolytica* and *P*. *multocida*). A limitation of this study was the absence of *Mycoplasma bovis* in the assay specificity analysis, an agent recognized as an important pathogen involved in BRD.

Before testing clinical samples from animals suspected of BRD, we investigated the interference of the biological matrix in our assay. We spiked negative clinical nasal swab samples with the previously quantified BRD viral agents. We demonstrated that the biological matrix usually submitted to diagnosis (nasal/respiratory secretions) had no influence on the analytical sensitivity of the assay. Indeed, the LoDs of the assay in spiked samples were the same of those observed when virus suspensions amplified in cell culture were used.

Using 289 clinical samples from the respiratory tract of cattle with clinical presentation compatible/suspected of BRD, we successfully achieved amplification of at least one viral target in 43 samples (14.8%), with BPIV-3 being the most frequently detected target (19/43). Although most samples were nasal swabs, the assay also investigated the viral targets in lung tissues (10/289), with detection of one BRSV positive sample. As the multiplex sensitivity and performance/results agreed with individual PCR/RT-PCR tests, these results reflect the real (or approximate) presence/frequency of the agents in these samples, discounting minor false negative results due to improper sample collection and/or conservation, or even the presence of the agents in amounts below our LoD. No viral co-detection was observed among the investigation agents in the analyzed samples. Although BRD is considered multifactorial syndrome, mixed infections frequently involve viral and bacterial pathogens rather than multiple viruses. Therefore, the absence of co-detection should be interpreted with caution, since bacterial pathogens were not investigated in the present study.

 Additional limitations of the present multiplex assay should be acknowledged. The assay is limited to the major viral BRD pathogens because of the restrict number of amplicons that can be differentiated by agarose gel electrophoresis. Another inherent limitation of endpoint multiplex assays is the inability to quantify viral loads, in contrast to qPCR assays.

In sum, we described a sensitive and specific endpoint multiplex PCR/RT-PCR to serve as an affordable alternative tool to diagnosis of the major viral agents associated with BRD. The assay would contribute for an easier, faster and more comprehensive etiological diagnosis of BRD, especially for laboratories lacking sophisticated and expensive qPCR equipment and platforms.

## Supplementary Information

Below is the link to the electronic supplementary material.

Supplementary Material 1 (DOCX 414 KB)

## Glossary

BRD: Bovine respiratory disease
BCoV: Bovine coronavirus
BoAHV-1: Bovine alphaherpesvirus 1
BRSV: Bovine respiratory syncytial virus
BPIV-3: Bovine parainfluenza virus 3
BVDV-1: Bovine viral diarrhea virus 1
BVDV-2: Bovine viral diarrhea virus 2
CAPES: Coordenação de Aperfeiçoamento de Pessoal de Nível Superior
CNPq: Conselho Nacional de Desenvolvimento Científico e Tecnológico
ICTV: International Committee on Taxonomy of Viruses
HoBiPeV: *Orthopestivirus brazilense*
*H. somni*: *Histophilus somni*
*M. haemolytica*: *Mannheimia haemolytica*
*P. multocida*: *Pasteurella multocida*
SV: Virology Section
UFSM: Universidade Federal de Santa Maria
USP: Universidade de São Paulo
