# Supplementary material for: An endpoint multiplex PCR/RT-PCR for detection of viral agents of bovine respiratory disease
Source: Braz J Microbiol. 2026 Nov 2;57(1):316. doi: 10.1007/s42770-026-02097-2 (PMC13728016; doi:10.1007/s42770-026-02097-2)
Supplement: Supplementary file 1 [file 42770_2026_2097_MOESM1_ESM.docx]

**Supplementary file**

**Fig. S1. Determination of the sensitivity of the multiplex PCR/RT-PCR.** Detection limit of the multiplex assay for BPIV-3 (a), BoAHV-1 (b), BVDV (c), BRSV (d), and BCoV (e). The viruses were titrated in TCID_50_ and subjected to tenfold serial dilutions. For BCoV, the detection limit was determined using a pool of five positive samples subjected to tenfold serial dilutions. M50: 50bp DNA Ladder (marker) (Ludwig, RS, Brazil). NC: negative control.
